# Seroepidemiology of *Leptospira* serovar Hardjo and associated risk factors in smallholder dairy cattle in Tanzania

**DOI:** 10.1371/journal.pntd.0011199

**Published:** 2023-04-05

**Authors:** Shabani Kiyabo Motto, Luis E. Hernandez-Castro, Gabriel Mkilema Shirima, Isaac Joseph Mengele, Shedrack Festo Bwatota, Barend Mark de Clare Bronsvoort, Eliamoni Titus Lyatuu, Daniel Mushumbusi Komwihangilo, Elizabeth Anne Jessie Cook

**Affiliations:** 1 Department of Global Health and Bio-Medical Sciences, School of Life Science and Bio-engineering, The Nelson Mandela African Institution of Science and Technology, Arusha, Tanzania; 2 Tanzania Veterinary Laboratory Agency, Central Veterinary Laboratory, Dar es Salaam, Tanzania; 3 The Roslin Institute, University of Edinburgh, Easter Bush, The United Kingdom; 4 Tanzania Veterinary Laboratory Agency, Dodoma, Tanzania; 5 Centre for Tropical Livestock Genetics and Health, The Roslin Institute, University of Edinburgh, Easter Bush, The United Kingdom; 6 International Livestock Research Institute, Mwenge Coca-Cola Road, Mikocheni, Dar es Salaam, Tanzania; 7 Tanzania Livestock Research Institute, Dodoma, Tanzania; 8 International Livestock Research Institute, Nairobi, Kenya; 9 Centre for Tropical Livestock Genetics and Health, International Livestock Research Institute, Nairobi, Kenya; University of Kentucky College of Medicine, UNITED STATES

## Abstract

**Background:**

Smallholder dairy farming is crucial for the Tanzanian dairy sector which generates income and employment for thousands of families. This is more evident in the northern and southern highland zones where dairy cattle and milk production are core economic activities. Here we estimated the seroprevalence of *Leptospira* serovar Hardjo and quantified potential risk factors associated with its exposure in smallholder dairy cattle in Tanzania.

**Methods:**

From July 2019 to October 2020, a cross-sectional survey was carried out in a subset of 2071 smallholder dairy cattle. Information about animal husbandry and health management was collected from farmers, and blood was taken from this subset of cattle. Seroprevalence was estimated and mapped to visualize potential spatial hotspots. The association between a set of animal husbandry, health management and climate variables and ELISA binary results was explored using a mixed effects logistic regression model.

**Results:**

An overall seroprevalence of 13.0% (95% CI 11.6–14.5%) for *Leptospira* serovar Hardjo was found in the study animals. There was marked regional variations with the highest seroprevalence in Iringa 30.2% (95% CI 25.1–35.7%) and Tanga 18.9% (95% CI 15.7–22.6) with odds ratios of OR = 8.13 (95% CI 4.23–15.63) and OR = 4.39 (95% CI 2.31–8.37), respectively. Multivariate analysis revealed the individual animal factors that were a significant risk for *Leptospira* seropositivity in smallholder dairy cattle were: animals over 5 years of age (OR = 1.41, 95% CI 1.05–1.9); and indigenous breed (OR = 2.78, 95% CI 1.47–5.26) compared to crossbred animals SHZ-X-Friesian (OR = 1.48, 95% CI 0.99–2.21) and SHZ-X-Jersey (OR = 0.85, 95% CI 0.43–1.63). Farm management factors significantly associated with *Leptospira* seropositivity included: hiring or keeping a bull for raising purposes (OR = 1.91, 95% CI 1.34–2.71); distance between farms of more than 100 meters (OR = 1.75, 95% CI 1.16–2.64); cattle kept extensively (OR = 2.31, 95% CI 1.36–3.91); farms without cat for rodent control (OR = 1.87, 95% CI 1.16–3.02); farmers with livestock training (OR = 1.62, 95% CI 1.15–2.27). Temperature (OR = 1.63, 95% CI 1.18–2.26), and the interaction of higher temperature and precipitation (OR = 1.5, 95%CI 1.12–2.01) were also significant risk factors.

**Conclusion:**

This study indicated seroprevalence of *Leptospira* serovar Hardjo, as well as the risk factors driving dairy cattle leptospirosis exposure in Tanzania. The study showed an overall high leptospirosis seroprevalence with regional variations, where Iringa and Tanga represented the highest seroprevalence and risk. The study highlighted the urgent need to understand the human exposures and risks from this important zoonosis to develop control measures and awareness of the problem and quantify the economic and production impacts through abortion and milk loss. In addition, given that the available data was limited to *Leptospira* serovar Hardjo, the study recommends more studies to identify serologically the most common serovars in cattle for targeted vaccination and risk reduction.

## Author summary

Dairy production in Tanzania constitutes traditional cattle meat-milk, improved smallholder dairy and commercial dairy farms. Despite the slow growth of the sector, smallholder dairy farming system remained a crucial for income generation and employment for thousands of families. This is more evident in the northern and southern highland of Tanzania where over 70% improved dairy cattle and milk production are core economic activities. Although the proportion of improved dairy cattle is relatively small compared to indigenous cattle, improved dairy sector contributes to 30% of milk produced in Tanzania. Constrains of leptospirosis in dairy, particularly of serovar Hardjo, remain a problem of its ability to cause abortion and reduce milk production in many farms worldwide. For many years epidemiological surveillance of leptospirosis in Tanzanian dairy cattle population is limited. This study provides a current status of seroprevalence and driven risk factors of leptospirosis occurrence in smallholder dairy cattle population from six regions of Tanzania as well as mapping hotspot areas at the district administrative level.

## Introduction

Leptospirosis is a zoonotic disease caused by different serovars of *Leptospira spp*. The annual global human morbidity measured as Disability Adjusted Life Years (DALYs) is estimated to be 1.3 million and annual mortality is 580,000 people [1]. As a result, leptospirosis has been declared a worldwide public health disaster with the highest prevalence in tropical and subtropical countries where cases increase mainly during the wet season [2–4]. People who work in particular dairy farming systems can contract leptospires via skin cuts, abrasions and mucous membranes after exposure to contaminated urine, reproductive fluids, manure, mud or pasture [5]. While animals acquire infection through sharing pasture or water contaminated with urine from infected animals.

Currently, over 300 serovars have been identified, many of them are pathogenic in humans and animals [6–8]. The clinical presentation varies depending on animal immunity and serovar type with possible asymptomatic cases in livestock [9,10]. Specifically, *Leptospira* serovar Hardjo (*L*. *interrogans* and *L*. *borgpetersenii*) causes reproductive complications (stillbirth, abortion, infertility, and death) in cattle [11].

In Tanzania, leptospirosis is a major public health issue and many studies have reported seropositive cases or active *Leptospira* infections in humans, domestic and wild animals [12–16]. The earliest evidence of leptospirosis was documented in the late 1990s when *L*. *interrogans* serovar Hardjo was confirmed [14] for the first time in livestock as well as in people. *Leptospira* serovar Hardjo seropositivity of 15.0% has been reported both in traditional and smallholder dairy herds in Tanga [17], with an additional study showing 3% seropositivity for *Leptospira* serovar Hardjo in at risk occupational groups in the same region [18]. Similarly, a study conducted in Katavi region reported *Leptospira* serovar Hardjo seropositivity of 17.59% in cattle and 15.73% in humans [19].

The dairy production system in Tanzania consists of three sectors: traditional cow meat-milk, improved small-holder dairy and commercial dairy farms [20]. Although the proportion of improved dairy cattle is relatively small compared to indigenous cattle (2.5% of total cattle number), the improved dairy sector contributes to 30% of milk produced in Tanzania [21]. The southern highlands and northern part of Tanzania have about 70% of improved dairy cattle and are core milk-producing areas in the country [21]. Over 90% of these are grouped into smallholder dairy farmers settled across rural and peri-urban areas [22]. Previous work has indicated that more than 90% comprises smallholder dairy cattle farms keeping one to five cows, and practicing intensive farming system on 1–2 hectares in southern and northern part of Tanzania [21,23]. A recent review of leptospirosis epidemiology in Tanzania [24] demonstrated that surveillance of *Leptospira* serovars is lacking in many areas, particularly in dairy cattle. Despite the importance of *Leptospira* serovar Hardjo in livestock health and productivity as well as its potential to cause abortion, little effort has been made on investigating disease prevalence in dairy cattle and risks factors for exposure.

## Materials and methods

### Ethics statement

Ethics of the study for animal subjects was reviewed and approved by the International Livestock Research Institute Institutional Animal Care and Use Committee (ILRI-IACUC2018-27) and the research permit was granted by the Tanzania Commission for Science and Technology (COSTECH), Ref. (2019-207-NA-2019-95). Written consent forms were signed by cattle owners before the interview and sample collection. The qualified Livestock Field Officer (LFO) restrained the animals during sampling. Local approval was sought from regional and local government authorities (LGAs) under the Ministry of Livestock and Fisheries (MLF).

### Area of study

**Fig 1:**
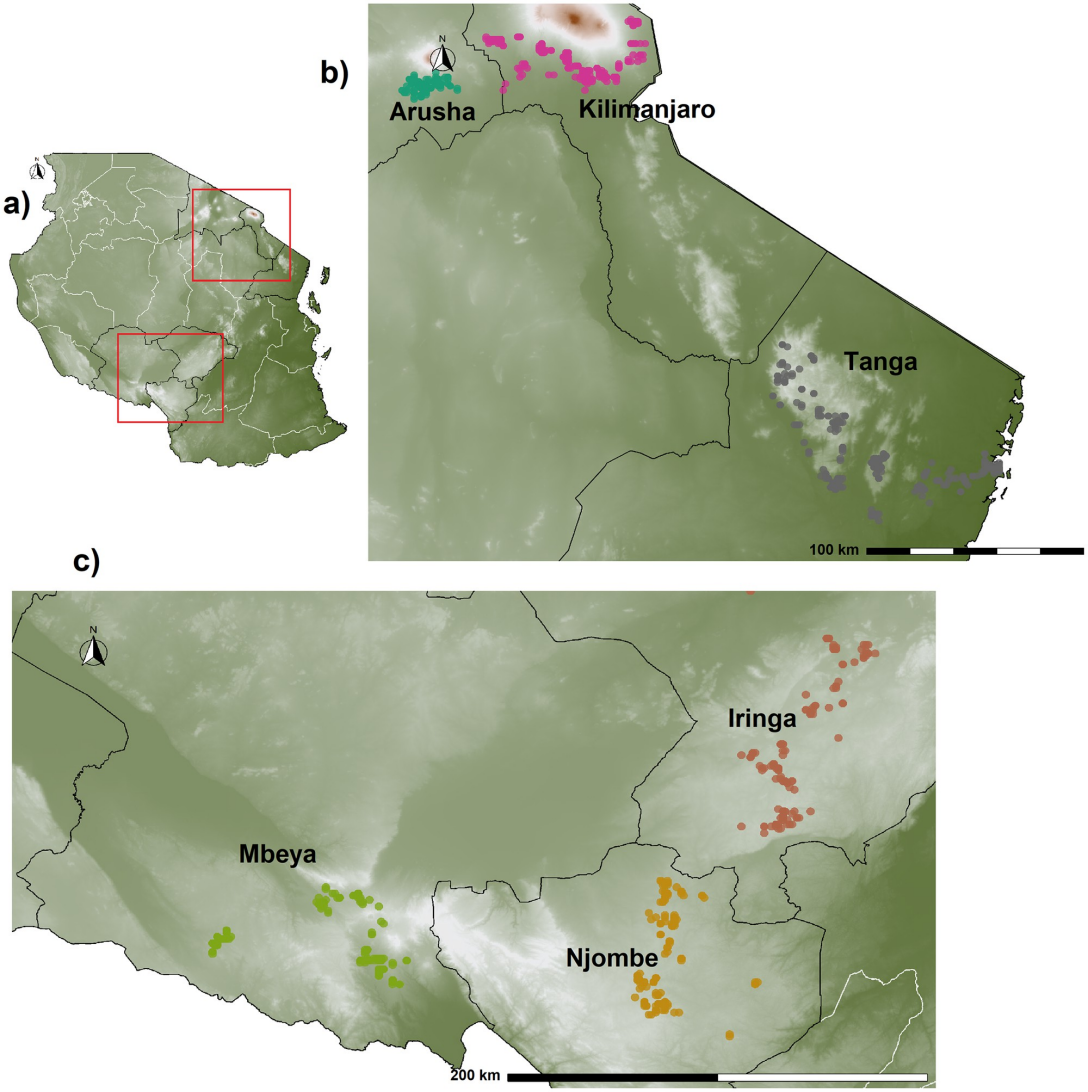
Geographic location of farms, regions, and dairy zones in Tanzania. **a)**, geographic location across six regions from two economically important dairy zones over an elevation map of Tanzania. Red squares indicate the important dairy zones. **b)**, a close-up of the northern zone integrated by the regions of Arusha, Kilimanjaro and Tanga in which a total of 12 districts were sampled. **c)**, a close-up of the southern highland zone of Tanzania integrating the Iringa, Njombe, and Mbeya regions in which 11 districts were sampled. In all panels, farm location (dots) is colour-coded to indicate their administrative region. Map source: https://www.usgs.gov/centers/eros/science/usgs-eros-archive-digital-elevation-global-multi-resolution-terrain-elevation.

Two key geographical zones (Fig 1a) representing 70% of the total improved dairy cattle across the country were chosen in this study [21]. The northern zone included the regions of Kilimanjaro, Arusha and Tanga (Fig 1b), whereas the southern highland zone was mainly formed by the Iringa, Njombe and Mbeya regions (Fig 1c).

### Study design

A cross-sectional study was carried out from July 2019 to October 2020. The cattle population in this study was selected from a subset of the cattle registry of the Africa Dairy Genetics Gains (ADGG) (https://data.ilri.org/portal/dataset/adgg-tanzania) program. Cattle (n = 50,000) had previously been enrolled in the ADGG program and smallholder dairy farmers participated in monthly data collection activities related to animal production. Of these 4000 cattle had known genetic characteristics and could be identified by their preliminary information such as an ear tag number, age, and sex.

For possible leptospirosis risk factors in smallholder dairy farming, we designed a questionnaire survey which was uploaded to the Open Data Kit (ODK) cloud platform software (https://getodk.org) version 1.22.4, and accommodated in Android device. The farm owner or animal caretakers were interviewed, and their answers were recorded onto the ODK form. The information collected included demographic and herd management details, animal health data, vaccination practices, water sources, and presence of rodents, dogs, cats or pigs on the farm or neighbouring farms. Additionally, geographic coordinates of each farm were recorded to map the seropositive animals and farms after laboratory testing. Final forms were transferred via secure network connection, and aggregated on the server at ILRI, Nairobi, Kenya prior to analysis.

### Serology sample

A blood sample was collected from the jugular vein into a 10ml blood collection tube (BD Vacutainer with no additives). Tubes were barcoded, labelled with date, animal identification number, and the barcode was scanned into the ODK survey form to link the animal biodata and the farm/herd owners. While in the field, samples were allowed to clot in a cool box filled with icepacks. Serum was prepared in the laboratory and stored at -20°C before testing as previously described [19].

### Leptospira ELISA

The Linnodee *Leptospira* Hardjo ELISA Kit (Linnodee Animal Care, Oakmount, Holestone Road, Ballyclare, Northern Ireland BT39 0TJ) was used to test sera for the presence of antibodies against lipopolysaccharide (LPS) epitopes that are found on *Leptospira* serovar Hardjo envelope [11,25]. Test sera were added to a 96 well-plate along with positive and negative controls provided in the kit and the test run as previously described [11]. Finally, the optical density (OD) was measured at 450nm using the Synergy HTX Multi-Mode Microplate Reader (BioTek Instrument, Inc. Highland Park, Winooski, VT 05404–0998) and used to calculate the positivity ratio (PR).


$$PR=\frac{Mean\;sample\;OD-Mean\;negative\;control\;OD}{Mean\;positive\;control\;OD-Mean\;negative\;control\;OD}$$

The sensitivity and specificity of this ELISA have previously been reported to be 100% and 86.67%, respectively [26].

### Statistical analyses

Seroprevalence estimates were calculated by dividing the number of positive samples by the number of cattle sampled. We also calculated an adjusted seroprevalence accounting for the stratified sampling design using *svydesign* functions in the *survey* R package [27]. Weights for each region were calculated by dividing the cattle population in each region by the number of sampled cattle [28].

We performed univariable analyses in the *epitools* R package [29] to measure associations at animal level, environmental and farm management variables (age, sex, breed, region, water source, herd size, abortion, multiple farm milking practices, hiring bull for breeding, presence of rodent in farm, grazing system, farm to farm distance, education and training by the farmer, farmer’s gender, experience in dairy farming, disposal of aborted/placental material, animal body condition score, animal contact with pigs and cat) and the binary ELISA results. Additional environmental data such as population density and solar radiation were sourced from the open.africa, elevation map on USGS, land cover on CCI Land Cover LC, and the mean annual temperature, precipitation from worldclim.org. To avoid multicollinearity, the Spearman’s rank correlation coefficient (rho) and Pearson tests were run on continuous environmental variable pairs to ensure they were uncorrelated (rho < 0.29 based on Cohen [30].

All variables with significance (*p* < 0.05) association in univariable analyses and uncorrelated continuous variables were further considered for multivariable risk factors analyses. To model the relationship between our ELISA binomial results and a set of covariates, we built a binomial (logistic) generalised mixed effects model with a logit link function implemented in the template model builder *glmmTMB* package [29]. Model selection was a backward stepwise approach where all significant variables (*p < 0*.*05*) from univariable analysis and continuous environmental variables were included in the initial model and eliminated one at time. Nested models were compared using the Akaike Information Criterion (AIC) and those models with the lowest AIC were kept until the end. When two nested models had a very similar AIC, likelihood ratio tests allowed us to identify the best model (*X*^*2*^ statistic *p* < 0.05; see S1 Table). Further, a final model was assessed by simulating residuals using the *simulateResiduals* function from the *DHARMa* package and estimating the amount of variance explained by the model (marginal and conditional R^2^). The model was considered efficient if residuals were plotted versus fitted values and each fixed effect showed no clear pattern.

## Results

### Descriptive results

A total of 2086 out of 4000 animals were sampled from 1370 dairy farms. The reduced number of animals was due to animals being sold, slaughtered or having died. Of these 2086, 15 animals were excluded since they could not be linked to the main ADGG animal registry. The total number of animals sampled per region was Tanga (n = 523), Kilimanjaro (n = 520), Arusha (n = 318), Iringa (n = 305), Mbeya (n = 218), and Njombe (n = 187). The mean age of the sampled cattle was 5.5 years. Of the farms visited, the average animal per herd was 2 and animals were mostly (97.3%) clinically healthy females without udder or reproductive complications. Sampled animals were categorized into four breed types based on their records from the ADGG cattle registry. There were three crossbreed groups including crosses of shorthorn zebu (SHZ) with European breeds such as Friesian (SHZ-X-Friesian), Ayrshire (SHZ-X-Ayrshire) and Jersey (SHZ-X-Jersey), and the fourth group included all indigenous/local breeds. The highest number of animals were SHZ-X-Friesian (n = 1415), followed by SHZ-X-Ayrshire (n = 433), SHZ-X-Jersey (n = 144), and indigenous breed (n = 79). Over 80% of the farms were close to the neighbouring farm (within 100 meters) demonstrating intensive farming system with few herds practicing extensive pasture grazing system (distance between farms 100–500 meters). Our environmental data set showed a mean annual temperature and precipitation of 19.9°C and 1238mm, respectively; however slightly variations were present between regions. No farms reported vaccinating against *Leptospira* or any other preventative measures to *Leptospira* infection.

### Seroprevalence

Of the 2071 animal sera tested, 269 (13.0%, 95% CI 11.6–14.5%) had antibodies against *Leptospira* serovar Hardjo. The adjusted seroprevalence accounting for the study design and differences in regional population sizes was 7.9% (95% CI: 3.9–11.8%).

The seropositivity was significantly related to breed with a high proportion of indigenous cattle being seropositive, 38.0% (95% CI 27.3–49.6%) compared to 12.7% (95% CI 11.0–14.6%) in SHZ-X-Friesian, 11.1% (95% CI 6.5–17.4%) in SHZ-X-Jersey, and 9.9% (95% CI 7.3–13.1%) in SHZ-X-Ayrshire (Table 1).

**Table 1:** Univariable associations between *Leptospira* serovar Hardjo seropositive results in dairy cattle and a set of variables. Independence test (fisher exact) two-sided p-values (P-value) is provided for each level.

Variables	Positive animal	Total animal	Prevalence (%), 95% CI	OR, 95% CI	p.value
**Breed type**					
SHZ-X-Ayrshire	43	433	9.93, 7.28–13.14	Ref	
SHZ-X-Jersey	16	144	11.11, 6.49–17.42	1.13, 0.62–2.08	0.75
SHZ-X-Friesian	180	1415	12.72, 11.03–14.57	1.32, 0.93–1.88	0.13
Indigenous	30	79	37.97, 27.28–49.59	5.55, 3.19–9.65	0.001
**Animal sex**					
female	251	2007	12.51, 11.09–14.03	Ref	
male	18	64	28.13, 17.6–40.76	2.74, 1.56–4.80	0.001
**Animal age** *					
≤ 5 years	118	1177	10.03, 8.37–11.88	Ref	
> 5 years	151	891	16.95, 14.54–19.58	1.83, 1.41–2.37	0.001
**Abortion in last 12 months**					
no	227	1881	12.07, 10.63–13.63	Ref	
yes	42	190	22.11, 16.42–28.68	2.07, 1.43–2.99	0.001
**Herd size**					
≤ 2	73	871	8.38, 6.63–10.42	Ref	
> 2	196	1200	16.33, 14.28–18.55	2.13, 1.61–2.84	0.001
**Livestock training**					
no	152	1444	10.53, 8.99–12.22	Ref	
yes	117	627	18.66, 15.68–21.93	1.95, 1.5–2.53	0.001
**Breeding method**					
use AI	136	1508	9.02, 7.62–10.58	Ref	
keep/hire bull	133	563	23.62, 20.17–27.35	3.12, 2.40–4.06	0.001
**Feeding system**					
intensive	171	1767	9.68, 8.34–11.15	Ref	
extensive	98	304	32.24, 27.01–37.81	4.44, 3.33–5.92	0.001
**Water source**					
Tap	143	1319	10.84, 9.21–12.65	Ref	
Well	126	752	16.76, 14.15–19.62	1.66, 1.28–2.14	0.001
**Distance between farms**					
≤ 100m	135	1511	8.93, 7.54–10.49	Ref	
> 100m	134	560	23.93, 20.45–27.68	3.21, 2.47–4.17	0.001
**Farmer with cats in the farm** *					
yes	224	1882	11.9, 10.47–13.45	Ref	
no	45	183	24.59, 18.54–31.49	2.41, 1.68–3.47	0.001
**Gender based farm management**					
female	92	835	11.02, 8.97–13.34	Ref	
male	177	1236	14.32, 12.41–16.4	1.35, 1.03–1.77	0.03
**Education**					
primary or none	144	1516	9.5, 8.07–11.09	Ref	
post primary	125	555	22.52, 19.11–26.23	2.77, 2.13–3.6	0.001
**Region**					
Mbeya	11	218	5.05, 2.55–8.85	Ref	
Kilimanjaro	26	520	5, 3.29–7.24	0.99, 0.48–2.04	1
Arusha	25	318	7.86, 5.15–11.39	1.61, 0.77–3.34	0.22
Njombe	16	187	8.56, 4.97–13.52	1.76, 0.8–3.89	0.17
Tanga	99	523	18.93, 15.66–22.55	4.39, 2.31–8.37	0.001
Iringa	92	305	30.16, 25.06–35.65	8.13, 4.23–15.63	0.001

* indicates where variables are not equal to 2071 due to missing data, **OR** = Odd ratio, **CI** = Confidence interval, **AI** = Artificial insemination

There was marked regional variation with the highest seroprevalence in Iringa Region 30.2% (95% CI 25.1–35.7%) and Tanga Region 18.9% (95% CI 15.7–22.6%).

**Fig 2:**
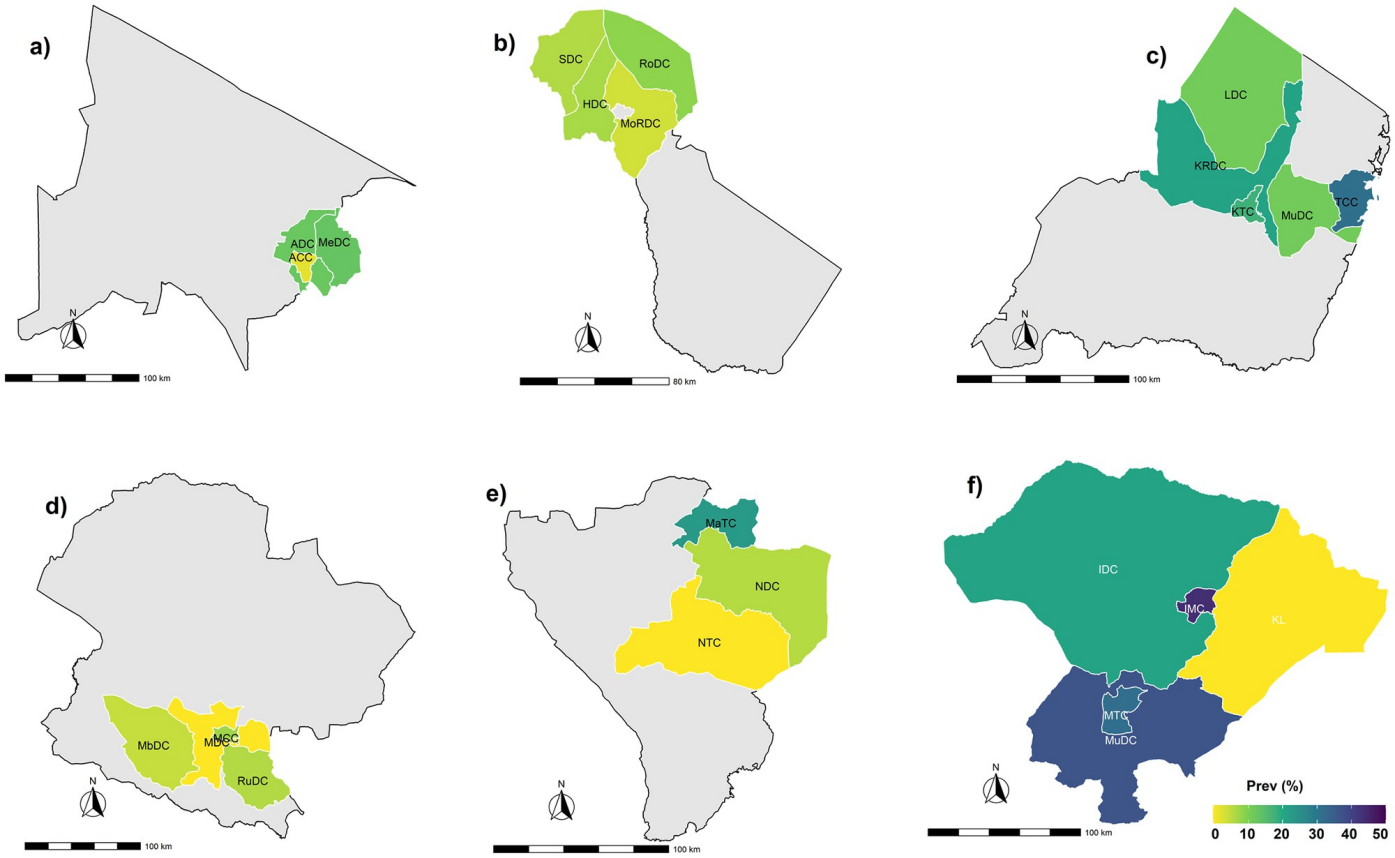
Geographic mapping of leptospirosis distributions and hotspots in 24 districts of study across six regions from two economically important dairy zones of Tanzania. The northern zone (**a**, **b**, and **c**) and southern zone (**d**, **e**, and **f**). **a)** Arusha region consisting of Arusha City Council (ACC), Meru District Council (MeDC), Arusha District Council (ADC), **b)** Kilimanjaro region consisting of Rombo District Council (RoDC), Moshi District Council (MoRDC), Hai District Council (HDC), Siha District Council (SDC), **c)** Tanga region consisting of Tanga City Council (TCC), Muheza District (MuDC), Korogwe District (KRDC), Korogwe Town Council (KTC), Lushoto District (LDC), **d)** Mbeya region consisting of Mbeya District Council (MDC), Mbeya City Council (MCC), Mbozi District Council (MbDC), **e)** Njombe region consisting of Njombe District Council (NDC), Makambako Town Council (MaTC), Rungwe District Council (RuDC), Njombe Town Council (NTC) and **f)** Iringa region consisting of Iringa District Council (IDC), Iringa Municipal Council (IMC), Mafinga Town Council (MTC), Mufindi District Council (MuDC). Map source: data shape file for Tanzania map at all levels downloaded from https://gadm.org/.

The spatial distribution and leptospirosis hotspots in dairy cattle at the district administrative level in the six regions of northern and southern part of Tanzania are demonstrated in Fig 2. Briefly, in Iringa Region the following districts were identified as hotspots, Mufindi District Council, Iringa Municipal Council, and Mafinga Town Council, and in Tanga Region, Tanga Town Council, and Korogwe District Council were identified as hotspots for seropositive cattle.

### Potential risk factors

The univariable analysis was performed with twenty-five variables at the initial screening. However, fourteen variables grouped at animal level and farm management were identified significantly associated to leptospirosis occurrence in Tanzanian dairy cattle (p ≤ 0.05) which were included in multivariable analysis. Uncorrelated continuous environmental variables (temperature and precipitation) were also included in multivariable analysis.

The significant variables included animal level such as breed in which indigenous animals were significantly more likely to be seropositive than other breeds (OR = 5.55, 95% CI 3.19–9.65); male animals were more likely to be seropositive (OR = 2.74, 95% CI 1.56–4.80); animals aged over 5 years (OR = 1.83, 95% CI 1.41–2.37); and animals which had abortion in the previous 12 months (OR = 2.07, 95% CI 1.43–2.99).

Management factors that were significantly associated with leptospirosis seropositivity after univariable analysis were herd size greater than 2 animals (OR = 2.13, 95% CI 1.61–2.84); breeding method by keeping or hiring bull from neighbouring farm (OR = 3.12, 95% CI 2.40–4.06); extensive grazing on pasture versus intensive zero grazing farming system (OR = 4.44, 95% CI 3.33–5.92); keeping cats against no cat in the farm (OR = 2.41, 95% CI 1.68–3.47); livestock farmers with training on livestock husbandry (OR = 1.95, 95% CI 1.5–2.53); well or river water sources (OR = 1.66, 95% CI 1.28–2.14).

In the final model, we included eleven fixed effects (that is, breed, animal age, livestock training, breeding method, feeding system, distance between farms, farm cat, region, temperature, precipitation and the interaction between temperature and precipitation) and incorporated the dependency among observations by using District, α, as a random effect.

$$Yij\sim Bin\left(1,pij\right)$$

$$E\left(Yij\right)=\sim \left(pij\right)$$

$$\text{logit}\left(pij\right)=\alpha +\beta_{1}\;\text{x}\;{\text{breed}}_{ij}+\beta_{2}\;\text{x}\;{\text{animal}\;\text{age}}_{ij}+\beta_{3}\;\text{x}\;{\text{livestock}\;\text{training}}_{ij}+\beta_{4}\;\text{x}\;{\text{breeding}\;\text{method}}_{ij}+\beta_{5}\;\text{x}\;{\text{feeding}\;\text{system}}_{ij}+\beta_{6}\;\text{x}\;{\text{distance}\;\text{farms}}_{ij}+\beta_{7}\;\text{x}\;{\text{farm}\;\text{cat}}_{ij}+\beta_{8}\;\text{x}\;{\text{region}}_{ij}+\beta_{9}\;\text{x}\;{\text{temperature}}_{ij}+\beta_{10}\;\text{x}\;{\text{precipitation}}_{ij}+\beta_{11}\;\text{x}\;{\text{temperature}\;\text{X}\;\text{precipitation}}_{ij}\;\alpha_{i}$$

$$\alpha_{i}\sim N(0,\sigma^{2}{}_{\alpha })$$

Where, *Yij* is the *j*th ELISA result binomially distributed with a conditional probability, *pij*, in district *i*, and *i* = 1, 20, and district, α_*i*_, is the random intercept, which is assumed to be normally distributed with mean 0 and variance *σ*^*2*^. Model assumptions were not violated as shown in S1 Fig, and the model explained 29.1% of the variation (conditional R^2^) of which 5.9% was due to random effect.

**Fig 3:**
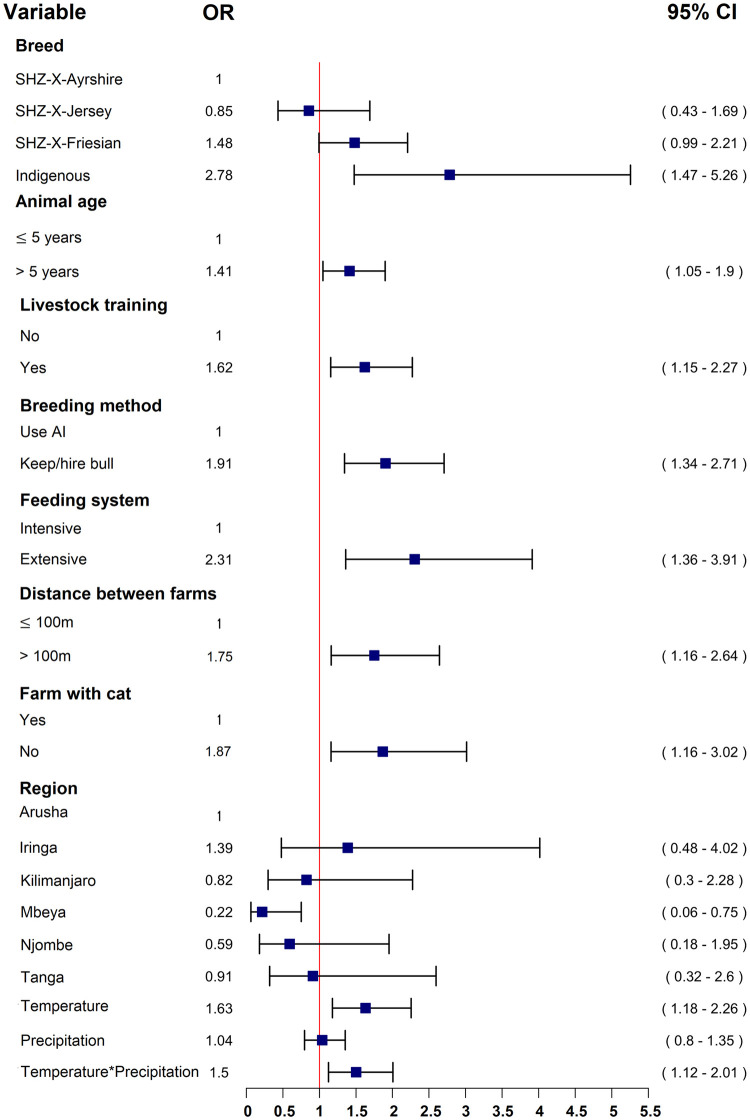
A forest plot summarizing the final multivariable model of significant predictive variables for leptospirosis association to seropositive occurrence in smallholder Tanzanian dairy cattle.

**Fig 4:**
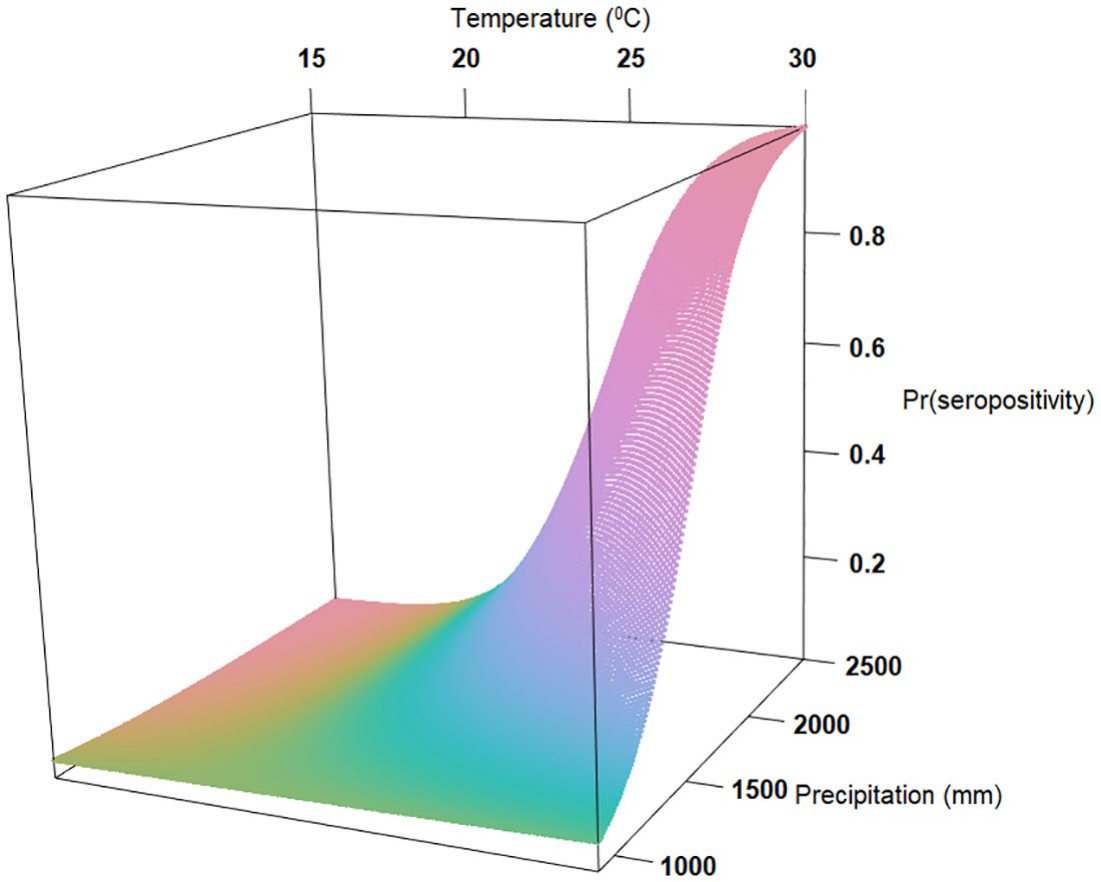
Three-dimensional graph shows the predicted probability of *Leptospira* serovar Hardjo seropositivity, Pr(seropositive), as a result of the interaction of increased temperature (°C) and precipitation (mm), and accounting for all other fixed effects in final generalised linear mixed effects model.

The identified risk factors for antibodies to *Leptospira* in cattle from the multivariable model (Fig 3) included: age equal to or over 5 years (OR = 1.41, 95%CI 1.05–1.9); Indigenous breed (OR = 2.78, 95%CI 1.47–5.26) compared to other breeds, farmers with livestock training (OR = 1.62 95%CI 1.15–2.27); hiring a bull for breeding (OR = 1.91, 95% CI 1.34–2.71), farm without cats (OR = 1.87, 95% CI 1.16–3.02), animals grazed extensively (OR = 2.31, 95% CI 1.36–3.91) and more than 100 meters distance between the farms (OR = 1.75, 95%CI 1.16–2.64). Increase in temperature (OR = 1.63, 95% CI 1.18–2.26), and the interaction between increased temperature and precipitation (OR = 1.50, 95%CI 1.12–2.01) were also found to be significant risk factors (Figs 3 and 4).

## Discussion

This study estimated the seroprevalence of antibodies to *Leptospira* serovar Hardjo and quantified risk factors for exposure in dairy cattle in Tanzania. Given the importance of dairy farming in Tanzania, this study provides important insights and highlights the need for action given the high seroprevalence and identified hotspots of this globally neglected zoonosis.

Here we report the seroprevalence of *Leptospira* serovar Hardjo across the major dairy keeping regions of the northern and southern highlands of Tanzania. To our best understanding and knowledge, this study is the first to describe *Leptospira* seroprevalence in dairy cattle in the Southern Highlands with no previous studies in Iringa or Njombe regions and a previous study in Mbeya reporting one seropositive case [14].

There was a variation in seroprevalence between the regions. For instance, Iringa and Tanga recorded higher seroprevalence than the other regions with (30.2%, 95% CI 25.1–35.7) and (18.9%, 95% CI 15.7–22.6), respectively, and there was significant more risk for cattle raised in Iringa region (OR = 1.39, 95% CI 0.48–4.02) than the other regions included in the study. This is in line with other previous seroprevalence estimates in cattle of 17.59% in Katavi [19] and 15% in Tanga [17], The study observed higher proportions of seropositive cattle from the southern zone 19.8% than the northern zone 11.4%.

The highest leptospirosis seroprevalences globally seem to be in areas characterized by a relatively warm, temperate environment and high precipitation which are essential factors for viable leptospiral maintenance and dissemination [31,32]. Our model (Fig 3) suggested the probability of seropositivity in smallholder dairy cattle increased with higher temperature (OR = 1.63, 95% CI 1.18–2.26). Interestingly, the probability of seropositivity increased significantly (OR = 1.5, 95% CI 1.12–2.01) when both temperature and precipitation increased (Figs 3 and 4). As shown elsewhere [33], leptospirosis outbreaks in our study sites are likely to occur more frequently during the warm rainy season.

Cattle grazed under extensive farming were significantly more likely to be seropositive than cattle from farms practicing intensive grazing (OR = 2.31, 95% CI 1.36–3.91). Similarly, cattle on farms with a distance between farms of more than 100 meters were at higher risk (OR = 1.75,95% CI 1.16–2.64) of being seropositive than farms with below 100 meters distance. Farm to farm distance was set to 100m, since the average smallholder farm in Tanzania is 1.2 hectares, this means that intensively managed animals are unlikely to have direct contact with each other or share resources [34]. It was observed during the study that farms with increased distance between farms had greater access to pasture and direct contact between animals of neighbouring farms. In addition to the farm management practices, low biosafety and biosecurity potentially put dairy cattle at higher risk of leptospirosis and spread in the herd. These findings complement past studies which concluded that extensive farming practices and co-grazing encourage pathogen transmission to susceptible animals [35] through contact with infected animals and access to contaminated pastures and water [17].

Meanwhile, varying sources have reported different susceptibility of cattle to leptospirosis infection based on the age class. Older animals are more likely to be seropositive than younger animals [36], In this study dairy cattle with age above 5 years were more likely to be seropositive (OR = 1.41, 95% CI 1.05–1.9) than younger animals aged 5 years and below as previously described [37]. It should be noted that none of the 1370 study farms had history of vaccination, treatment, or any control measure against leptospirosis. This suggests that the high seropositivity to *Leptospira* serovar Hardjo in older cattle may be due to the increased possibility of exposure to *Leptospira* in the environment, and also carrier animals in the same herd [11].

Livestock training was an important factor for seropositivity in dairy cattle. Cattle belonging to farmers who received livestock training were at higher risk of being seropositive (OR = 1.62, 95% CI 1.15–2.27) than cattle belonging to farmers who did not have training on livestock keeping. This was contrary to expectations that animals belonging to farmers who received training on dairy keeping would be at lower risk to contract leptospirosis as the farmers abide by farm management and precautionary measures to prevent disease spread. The higher risk may be attributed to the practice of farmers hiring untrained personnel to take care of the animals as it was observed during the study.

Livestock production in Tanzania remains a challenge particularly for smallholder dairy farmers. In this study, several smallholder dairy farmers relied on breeding with a bull, and a few of them used artificial insemination (AI) which was not easily accessible because of the limited expertise for this service. Cattle on farms with kept or hired bull for breeding were more likely to be seropositive than cattle on farms using AI methods for breeding purposes (OR = 1.69; 95% CI 1.15–2.48). Hiring a bull for breeding in Tanzanian dairy cattle was shown to be an important factor for disease spread in animals within the herd, and between neighbour farms through sexual contact [38]. It has been reported by previous authors [36], that hiring a bull or close contact between animals for calf raising is the most remarkable determinant for leptospirosis infection in smallholder dairy farms.

This study found cattle breed was significantly associated with seropositivity with indigenous cattle being significantly more likely to be leptospirosis seropositive (OR = 2.78, 2.48 (95% CI 1.47–5.26) than SHZ-X-Friesian (OR = 1.48, 95% CI 0.99–2.21) or other crossbreeds. This is contrary to findings in other regions where crossbred cattle have been reported to have higher seropositivity [39]. Further work is required to understand the increased seroprevalence in indigenous cattle in this setting and if this also relates to increased disease susceptibility.

This study found that farms that do not keep cats in the farm were significantly more likely to have seropositive cattle (OR = 1.87, 95% CI 1.16–3.02). Epidemiologically, rodents are mainly known for carrying different pathogenic *Leptospira* and contaminate pasture [38], consequently livestock may acquire leptospirosis infection during grazing [40]. Keeping of cats in the farm was likely to reduce the rodent numbers particularly in cow sheds, in the reserved pastures, or hay barns which could be a protective measure to reduce exposure of cattle to *Leptospira* pathogens.

The findings in this study underpin the importance of leptospirosis in dairy farms. The presence of *Leptospira spp*. in dairy farms has previously been attributed to environmental contamination from the reservoirs of the pathogen and dairy animals that share grazing pastures and the environment [41]. Infected animals can contaminate the environment with leptospires by excretion in urine which can remain infectious in the environment for a few weeks to a month [42]. Contamination of the environment is linked to spread via water sources or animal feeds that can be accessed by other animal species [43]. These may also become sources of infection to animal caretakers or slaughterhouse workers [44]. It has already been highlighted that leptospirosis infection in humans is largely dictated by its prevalence in livestock [45].

## Conclusion

This study provides an insight into the epidemiological status and exposure in smallholder dairy cattle raised across the country to *Leptospira* serovar Hardjo. In addition, all dairy cattle in Tanzanian smallholder farms were not vaccinated against leptospirosis. The findings highlight that the disease is prevalent in smallholder dairy cattle population and there were high levels of leptospirosis exposure in specific regions. The disease seropositivity of the studied dairy cattle was significantly associated with individual animal factors such as age and breed, as well as with management practices such as knowledge on animal husbandry, keeping cat for rodent control, breeding practices, and distance between the farms. Precipitation and temperature were also significant environmental risk factors in this cattle population.

### Recommendation

The limitation of this study is the focus on only *Leptospira* serovar Hardjo exposure in cattle by ELISA. We recommend further study to identify additional serovars that might be missed from the test and that may be circulating in Tanzania smallholder dairy cattle. It is important for the future studies to consider additional serotyping methods such as microscopic agglutination test [19] or molecular typing [46] to characterize more serovars. For example, due to the fact that these similar regions have records of intensive pig breeding as well as improved dairy cattle, the presence of pigs in or near cattle bomas or farms may increase chances of contact between pigs and dairy cattle and thus spread of *Leptospira* serovars to cattle such as Pomona, Australis, and Tarassovi serovar which are principally maintained by pigs [47].

Moreover, more studies should be carried out with a special focus on human leptospirosis especially in smallholder dairy farmers. The high prevalence of leptospirosis in cattle may play an important role in disease transmission to humans, particularly to livestock keepers and slaughterhouse workers [18]. Generally, individual and community education regarding the risks of leptospirosis disease and prevention measures is recommended to control and prevent the spread of this zoonotic disease.

## Supporting information

S1 Table Model selection results for the generalised linear mixed effect model for Leptospirosis serovar Hardjo in smallholder dairy cattle. The most strongly supported model is number 7. For each model, formula, Akaike information criterion (AIC), and Loglikelihood ratio test p-value (LRT p-value) are provided.
(DOCX)

S1 Fig Simulation plot of predictable variable to validate best fit of model for leptospirosis occurrence predictions in smallholder dairy cattle in Tanzania. (TIF)
