# Supplementary material for: Seroepidemiology of Leptospira serovar Hardjo and associated risk factors in smallholder dairy cattle in Tanzania
Source: PLoS Negl Trop Dis. 2023 Apr 5;17(4):e0011199. doi: 10.1371/journal.pntd.0011199 (PMC10075398; doi:10.1371/journal.pntd.0011199)
Supplement: S1 Table — The most strongly supported model is number 7. For each model, formula, Akaike information criterion (AIC), and Loglikelihood ratio test p-value (LRT p-value) are provided. (DOCX) [file pntd.0011199.s001.docx]

Supplementary Table 1. Model selection results for the generalised linear mixed effect model for Leptospirosis serovar Hardjo in smallholder dairy cattle. The most strongly supported model is number 7. For each model, formula, Akaike information criterion (AIC), and Loglikelihood ratio test p-value (LRT p-value) are provided

| Model | Formula | AIC | LRT p-value |
| --- | --- | --- | --- |
| 1 | lepto ~ breed + animal sex + animal age + abortion + herd size + livestock training *_+_* breeding method + feeding system + distance farms + farm cat + education + gender + region + **water*** + temperature + precipitation + temperature x precipitation + (1 \| district) | 1362.758 |  |
| 2 | lepto ~ breed + animal sex + animal age + abortion + herd size + livestock training *_+_* breeding method + feeding system + distance farms + farm cat + **education*** + gender + region + temperature + precipitation + temperature x precipitation + (1 \| district) | 1359.885 | 0.569 |
| 3 | lepto ~ breed + animal sex + animal age + abortion + herd size + livestock training *_+_* breeding method + feeding system + distance farms + farm cat + **gender*** + region + temperature + precipitation + temperature x precipitation + (1 \| district) | 1358.032 | 0.7021 |
| 4 | lepto ~ breed + animal sex + animal age + **abortion*** + herd size + livestock training *_+_* breeding method + feeding system + distance farms + farm cat + region + temperature + precipitation + temperature x precipitation + (1 \| district) | 1357.087 | 0.3044 |
| 5 | lepto ~ breed + animal sex + animal age + **herd size*** + livestock training *_+_* breeding method + feeding system + distance farms + farm cat + region + temperature + precipitation + temperature x precipitation + (1 \| district) | 1355.269 | 0.6696 |
| 6 | lepto ~ breed + **animal sex*** + animal age + livestock training *_+_* breeding method + feeding system + distance farms + farm cat + region + temperature + precipitation + temperature x precipitation + (1 \| district) | 1353.371 | 0.7496 |
| 7 | lepto ~ breed + animal age + livestock training *_+_* breeding method + feeding system + distance farms + farm cat + region + temperature + precipitation + **temperature x precipitation** + (1 \| district) | 1353.903 | 0.1115 |
| 8 | lepto ~ breed + animal age + livestock training *_+_* breeding method + feeding system + distance farms + farm cat + region + temperature + precipitation + (1 \| district) | 1359.913 | 0.004653 ** |
