# Supplementary figures and images for: Seroepidemiology of Leptospira serovar Hardjo and associated risk factors in smallholder dairy cattle in Tanzania
Source: PLoS Negl Trop Dis. 2023 Apr 5;17(4):e0011199. doi: 10.1371/journal.pntd.0011199 (PMC10075398; doi:10.1371/journal.pntd.0011199)

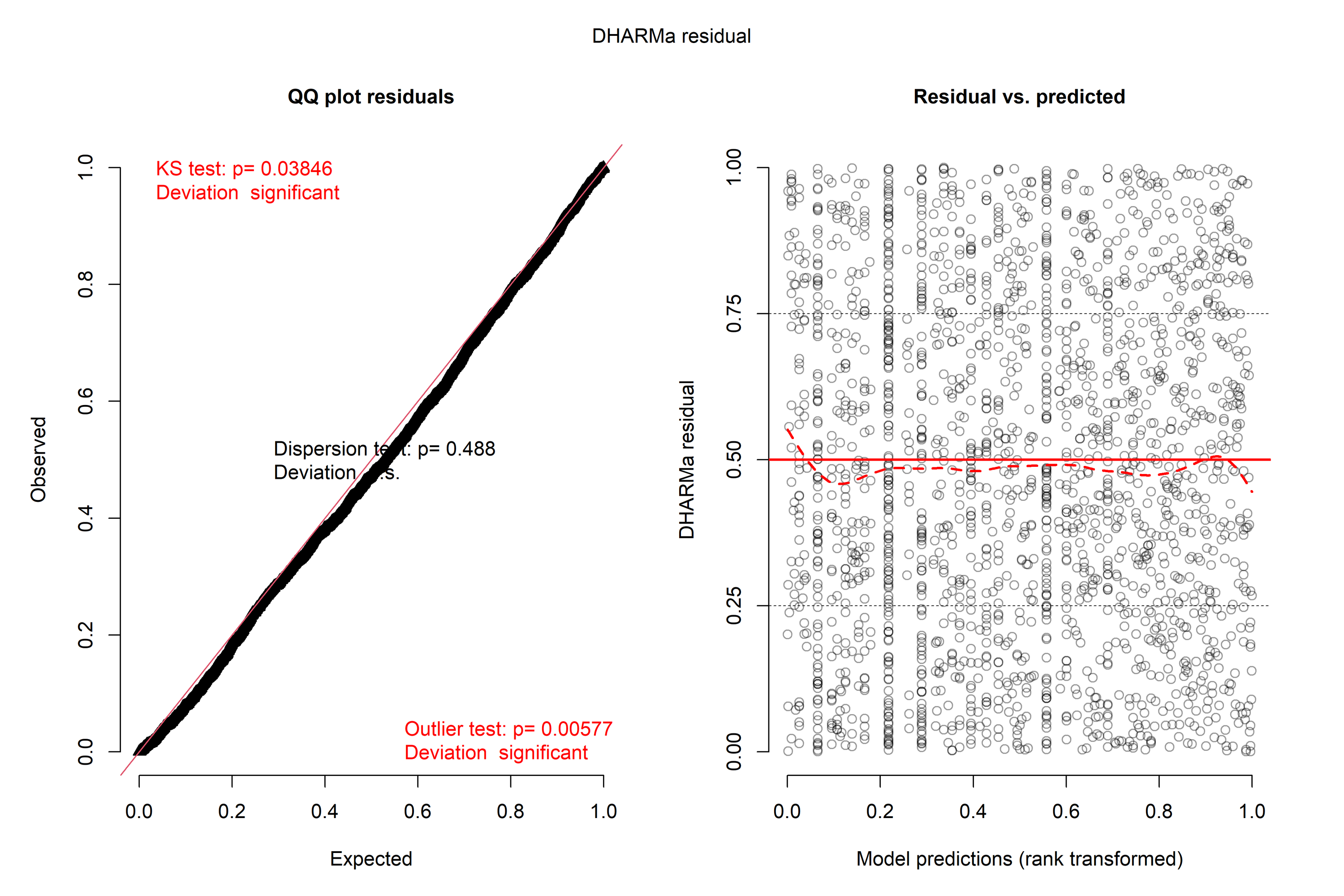

Supplement: S1 Fig — (TIF) [file pntd.0011199.s002.tif]
